# The potential for using smartphones as portable soil nutrient analyzers on suburban farms in central East China

**DOI:** 10.1038/s41598-019-52702-8

**Published:** 2019-11-11

**Authors:** Karolina Golicz, Stephen H. Hallett, Ruben Sakrabani, Genxing Pan

**Affiliations:** 1 School of Water, Energy and Environment, Cranfield University, Cranfield, Bedfordshire, MK43 0AL UK; 2 Department of Soil Science and Institute of Resource, Ecosystem and Environment of Agriculture, Nanjing Agricultural University, 1 Weigang, Nanjing, 210095 China

**Keywords:** Element cycles, Environmental monitoring

## Abstract

Soil testing is frequently conducted to specify nutrient supply recommendations. By adjusting fertilizer type and application rates, farmers can achieve desired crop yields with lower production costs and are thereby less likely to contribute to eutrophication of nearby waterbodies. However, traditional methods of soil testing can be costly, time-consuming and are often impractical in rural and resource-poor regions in China, where rapid population growth and consequent food demand must be balanced against potential environment risks. Smartphones are nearly ubiquitous and offer a ready capability for providing additional support for existing extension advice. In this study, we used an Android-based smartphone application, in conjunction with commercially-available Quantofix test strips, to analyze soil samples with a goal of providing specific fertilizer recommendations. The app transforms the smartphone into a portable reflectometer, relating the reaction color of the test strips to the concentration of soil nutrients available. A 6-month long field study involving two growing seasons of vegetables was conducted in a suburban area of Nanjing, Jiangsu Province of China to evaluate the accuracy and precision of smartphone-mediated soil analysis. Results obtained via the smartphone correlated well with the yield response of the common green vegetable *Ipomoea aquatica* (water spinach) and could be applied in calculations of necessary off-farm inputs throughout the open-field vegetable growing season. Together, the smartphone and test strip in combination were shown to offer an acceptable screening tool for soil nutrient concentration assessment with the potential to result in substantial monetary savings and reduction of nutrient loss to the environment.

## Introduction

Global food production has been greatly expanding to meet the growing world population, which is expected to reach 9.8 billion people by 2050. However, meeting these challenges introduces growing pressures on available land and water resources, often in combination with the effects of climate change impacts. Therefore, it becomes critical to boost food production sustainably, through adequate fertilizer supply. For the last 6 decades, issues of decreasing world soil fertility and imbalances in nutrient supply have been addressed by large-scale production and application of mineral fertilizers^1^. Global demand for mineral fertilizers (N + P_2_O_5_ + K_2_O) is growing annually by 1.9% and is expected to reach 201.66 million tonnes by the end of 2020^2^. However, high fertilizer inputs are often not translated into high resource-use efficiency, where the demand for nutrients is not met at the right spatio-temporal scale^3^. Studies have shown that plant uptake rates for N can be as low as 10–20% in horticultural systems^4^. Vegetable production, both in open land and greenhouse-based, involves frequent cultivation, high fertilizer application rates (up to 900 kg per ha), low rooting density and short growing-seasons – those systems are associated with high environmental risks of nutrient leaching and greenhouse gas emissions^5^. This is concerning given that the need for fresh produce, particularly vegetables, will increase alongside the awareness of the impact of poor nutrition on morbidity and mortality rates in relatively wealthy societies^6^. High input industrial monoculture practices should be considered low efficiency systems that are unlikely to produce enough food to feed the future world population whilst simultaneously absorbing the market shocks of volatile fossil fuel and fertilizer prices, and climate change-induced resource shortages.

Just as most developed countries must undertake coordinated efforts to sustainably transform their food systems, the developing nations must also take the opportunity to develop agroecologically efficient production techniques whilst building on the already available body of knowledge. Tittonell *et al*.^7^ compared maize yields of research-managed and farmer-managed plots and found that planting the crop early in the season with optimized planting densities, controlling pests/weeds and disease, and using hybrid seeds can together double the agriculture output of smallholder farms in western Kenya. Further improvements involving application of mineral fertilizers could increase the yields by +1 t ha^−1^, provided that there are measures put in place to improve fertilizer use efficiency^7^. One of the methods used to balance soil fertility with optimal farm output involves prescriptive-corrective crop nutrient management, where employment of monitoring procedures during crop growth enables adjustment of nutrient management practices to correct deficiencies or excesses^3^. In most developed nations, characterised by industrial-scale agriculture, farmers have access to the tools necessary for agricultural monitoring, such as laboratory tests of physico-chemical characteristics of soils and/or plant tissue^8^. Moreover, there are a number of inexpensive and useful field-based tools that can act as indicators of the soil fertility status^9^, and these include colorimetric test strips^10^. Such strips enable farmers to safeguard their businesses by optimising crop production whilst minimising financial and environmental risks arising from overfertilization.

By contrast, developing nations face challenges in accessibility to laboratory-based assessments of soil quality with common practices promoting the use of mineral fertilizers through blanket recommendations, based on region-wide soil surveying or on agroecological zoning, rather than being site and crop specific and accounting for small-scale heterogeneity in soil conditions^7^. In China, a national project “Soil testing for formulated fertilization” had been implemented since 2005, covering >90% of the total crop production area across the country. The implementation of the project has led to a reduction in chemical fertilizer use by approx. 3 Mt by 2009; increase in soil organic carbon and decrease in N fertilizer induced N_2_O emission from croplands^11^. However, the lack of access to technical services and difficulty with plot scale soil sampling and soil analysis have been identified as barriers to household farmers attaining benefits from such schemes^12^. There remains a need for cheap and accessible technologies that can act as an alternative to conventional plant tissue and soil testing. Smartphones used in conjunction with test strips offer such a technological opportunity because they: (1) are free of human bias associated with color detection; (2) are capable of providing precise and replicable results in contrary to the standard visual method; (3) have capacity for storing and geotagging results for future use, and; (4) offer the potential for inclusion of wider extension and agronomical advice alongside the immediate results, and; (5) offer a pragmatic alternative to expensive commercial reflectometers on offer by test strip manufacturers, such as the Quantofix Relax Reflectometer utilised with Quantofix test strips.

**Figure 1:**
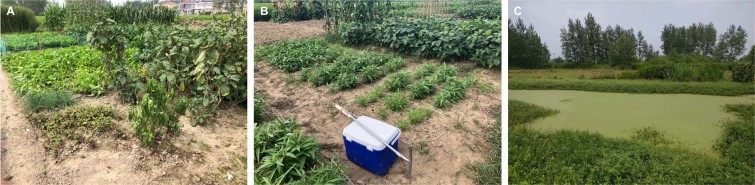
(**A**) Small-scale, multi-crop vegetable farms typical of suburban East China. (**B**) Study area subdivided into plots, (**C**) Nearby waterbody showing signs of eutrophication resulting from overfertilization.

In this study, we describe how one smartphone app, Akvo Caddisfly, being available via the Android Google Play Store (https://play.google.com/store/apps/details?id=org.akvo.caddisfly&hl=en), might be used as an in-field soil nutrient analyzer in suburban vegetable farm in China (Fig. 1). Akvo Caddisfly is an application that transforms a smartphone into a portable reflectometer that can then be used to relate the concentration of the nutrient to the intensity of the color of a commercially available test strip. The reading of the test strip made in the Akvo Caddisfly app is passed through a calibration equation based on a laboratory study that has correlated a widely accepted colorimetric method of NO_3_^–^ - N and Olsen-P assessment with results provided by the app. Such results offer considerably more precision than an assessment ‘by eye’ comparing against a coarse color chart. The purpose of the study was to investigate the accuracy and precision of results obtained in field conditions in the sub-tropical climate and the capacity for smartphone-mediated soil analysis to monitor changes in soil nutrient status that were then used in making fertilizer recommendations. By employing Akvo Caddisfly, it is possible to provide farmers, who might otherwise have had limited access to conventional soil testing, with a simple decision support tool that can provide information about the quantity of plant available nitrate and phosphorus in the soil.

## Materials and Methods

### Site description

A field experiment was conducted between June and September 2018 in a vegetable farm in a suburban village of Qinfeng (31°16′ N, 119°54′ E), Luhe District, Nanjing, China. The region has a sub-tropical monsoon climate (annual mean T_(°C)_ = 15.6; precipitation = 1001 mm). The topsoil chemical characteristics prior to the commencement of the experiment were: pH (soil: water) of 4.3, electrical conductivity (EC) of 0.34 dS·m^−1^, and total N, total C and SOM content of 2.07 g·kg^−1^, 20.2 g·kg^−1^ and 53.0 g·kg^−1^, respectively. The soil has a broad classification as a gleysol with gleyic, reducing conditions and a particle size distribution in the upper 25 cm equivalent to 5.4% sand, 42.5% silt and 52.1% clay.

### Experimental design

**Figure 2:**
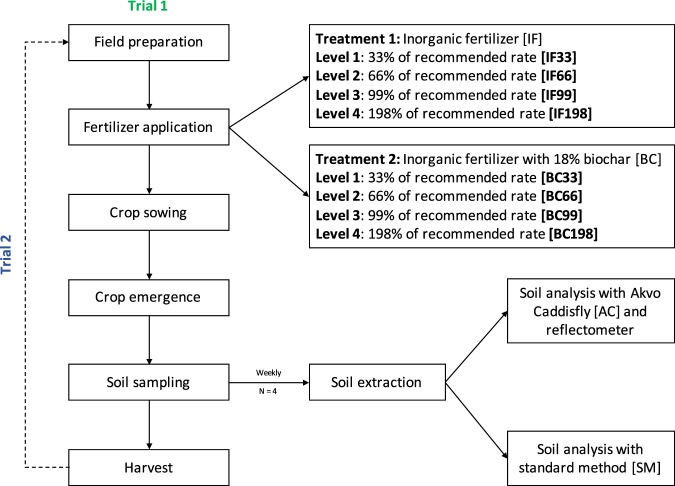
Flowchart showing the planning and execution of fieldwork activities. Field preparation involved ploughing and establishment of quadrant markings. There were two trials, which constituted two growth cycles of water spinach. Standard inorganic fertilizer (IF) and biochar-infused inorganic fertilizer (BC) was added as proportion of the recommended application rate. Soil sampling took place every week post crop emergence with 4 sampling events in a single growth cycle. Soil analysis was conducted immediately after sampling with standard methods and test strips assessed through Akvo Caddisfly and commercial grade reflectometer. Trial 2 commenced three weeks after the harvest of the first crop and involved the same procedures.

The experiment was performed with water spinach (*Ipomoea aquatica*) growing over two seasons with each season lasting for 35 days. The experiment had two treatments of fertilization scheme as a primary factor and N application rates as a secondary factor (Fig. 2). The two-fertilizer scheme involved application of normal compound inorganic fertilizer (15:15:15 NPK) and biochar organo-mineral fertilizer (15:15:10 NPK), containing 18% maize biochar. The four levels of N rates were respectively 33%, 66%, 99% and 198% of the recommended optimum N rate for water spinach, which was 136 kg ha^−1 ^^13^. Test plots were constructed in accordance with the Chinese raised-bed method; three permanent raised-bed plots were isolated from another via well-compacted paths. Stepping on the vegetable raised-bed plot was avoided, with weeding and watering activities taking place from the path. As the purpose of the experiment was not to accurately assess agronomic response of the crop to the fertiliser scheme used but to measure changes in the soil nutrient concentration via non-standard soil analytical methods in conditions likely to mimic those experienced in suburban Chinese farms, no further steps to isolate the plots were taken.

The experiment was organized in a randomized complete block design, comprising 36 blocks with four replications, each block in an area of 1.05 m^2^ (0.75 m × 1.40 m). Planting holes were located 20 cm from the edge of the block and set approx. 15 cm apart. Both plots and sub-plots (blocks) were clearly marked with bamboo field-markers and red tape stretching from marker to marker. Fertiliser was applied carefully within each block and incorporated into the soil in the centre of each plot. For Trial 1, the water spinach was planted on the 18^th^ of June and harvested on the 23^rd^ of July. Prior to the second fertilizer application, the fields were ploughed, and the quadrant markings re-established. For Trial 2, water spinach was planted again on the 16^th^ of August and harvested on the 20^th^ of September. The fertilizer was applied once prior to sowing and no herbicides or pesticides were applied. Daily management included irrigation and removal of weeds, manually by hand throughout the growing period. Hand irrigation was conducted with equipment available on the farm, between 6.30 and 7.30 AM daily, unless a rain event occurred in the previous 24-hour period.

### Data collection

Soil sampling was undertaken every seven days post seedling establishment, whereby 150 grams of soil were collected from each block. The soil was placed in a labelled sealable plastic bag and placed immediately in a portable cooling box. Subsequently, images of randomized blocks were taken following the procedure outlined by Easlon & Bloom^14^. Images were analysed to establish the total leaf area of the crop within each quadrant.

A portion of the soil was analysed for available N and extractable P using the Quantofix NO_3_^−^ and PO_4_^3−^ test strips and the Quantofix Relax Reflectometer and a Samsung Galaxy S8 mobile phone with the pre-installed Akvo Caddisfly app (Beta ver. 10). The soil was sieved with a 5.6 mm sieve and taken to an air-conditioned room for extraction and analysis (the sample temperature range throughout the experiment was 23–28 °C). The extracts were obtained by mixing 10 g of soil with 50 mL of distilled water (for nitrate) and 15 g of soil and 50 mL of Mehlich-1 solution (for phosphate) in 250 mL plastic bottles. The contents of the bottle were then shaken for a minimum of 5 minutes or until large blocks of soil (if present) were dissolved, the resultant mixture was then filtered through a Whatman no. 4 filter paper. Further dilution was applied where necessary, resulting in a clear extract used for testing. A test strip was wetted and placed on a color correction card to be analysed with Samsung Galaxy S8 after one minute of reaction time. Simultaneously, another test strip was wetted and passed through Quantofix Relax Reflectometer. The test strip-mediated soil analysis was conducted during daylight hours with an average of three test strip measurements per extract. The chief purpose of this study was to assess the viability of employing a smartphone as an in-field soil analyzer and thus, results obtained with Quantofix Relax Reflectometer will not be discussed further. In cases when nitrite was shown to be present in quantifiable amounts (≥1 mL L^−1^, as indicated by Akvo Caddisfly), it was neutralised with amidosulfuric acid (H_3_NSO_3_) with a ratio of 1 mL of 10% H_3_NSO_3_ to 5 mL of sample as to remove any effect associated with nitrite inference. Alongside the test strip measurements, the soil was analysed using standard laboratory techniques for comparison. For available N analysis, the soil was extracted with 2 M potassium chloride for 60 min on an orbital shaker (set at 180 rpm) with a soil to solution ratio 1:5 and determined colorimetrically following the standard method of Keeney and Nelson^15^. Available N analysis took place within 24 hours of sample collection. The remaining soil was air-dried and extracted with 0.5 M sodium hydrogen carbonate (pH: 8.5) for 30 min on an orbital shaker (180 rpm) with a soil to solution ratio 1:20 following the standard method of Murphy and Riley^16^. Extractable N and P analysis then took place via the Segmented Flow Auto-Analyzer (SKALAR).

The yield of each plot was obtained at harvest 35 days after planting. The harvest involved cutting water spinach at its base and transferring it to a clearly labelled plastic bag (one bag per 1.2 m^2^ quadrant). Plant fresh weight was measured immediately post-removal. Additionally, 1 kg of water spinach was dried in an oven at 65 °C for 72 hours to determine the dry weight of the harvested crop.

### Data processing and statistics

The results obtained via the standard and smartphone-mediated methods of soil analysis were multiplied by appropriate dilution factors and expressed as mg kg^−1^ and kg ha^−1^. Four data points collected via Akvo Caddisfly were discarded as the test strip was visibly discoloured as a result of chemical interferences. Statistical tests such as correlations and ANOVA were deemed inadequate for a study involving a method comparison. Instead, Bland-Altman (B-A) plots^17^ have been employed to investigate ﻿the degree of agreement and the absolute (Δ) difference between standard laboratory and smartphone-mediated methods of nutrient analysis. The B-A analysis involves constructing a scatter plot, in which the difference between the paired measurements is plotted on y-axis and average of the measures of two methods on x-axis. The mean difference refers to the bias between two methods and is represented as a central horizontal line on the plot. Two additional lines are derived from the standard deviation (SD) of differences between paired measurements and represent 95% limits of agreement (mean bias 1.96 SD). Analysis were carried out in R Studio (ver. 1.1.447) and the MethComp package. Fertilizer cost (5 CNY = £0.57) was established based on the amount of money the authors were charged in the local village shop. Cost savings were calculated to demonstrate saving potential for small (plot-scale) and large (1 ha field-scale) field sizes.

## Results and Discussion

### Plant response and residue soil nutrient content

The water spinach yield was strongly correlated with the fertilizer treatment for both standard inorganic fertilizer, IF, (Y = −5.941E − 5x^2^ + 0.0157 × +1.0292; R^2^ = 0.98) in Trial 1 and Trial 2 and inorganic fertilizer with 18% biochar, BC, (Y = −4.62E − 5x^2^ + 0.0159 × +1.0469, R^2^ = 0.95) and (Y = −4.631 − 5x^2^ + 0.014 × +0.074, R^2^ = 0.97; and Y = −6.333E − 5x^2^ + 0.0015 × +0.0812, R^2^ = 0.83, respectively). The vegetable yield was lower in Trial 2; this was considered likely to be due to lower rainfall and over-fertilization. Similar studies have noted a correspondingly high level of responsiveness of quick-growth green vegetables, including water spinach, to experimental treatment^18^. High residue nitrogen was recorded for treatments BC198 and IF198, which were equivalent to 272 kg of N per ha for Trial 1 and 334 kg of N per ha for Trial 2.

**Figure 3:**
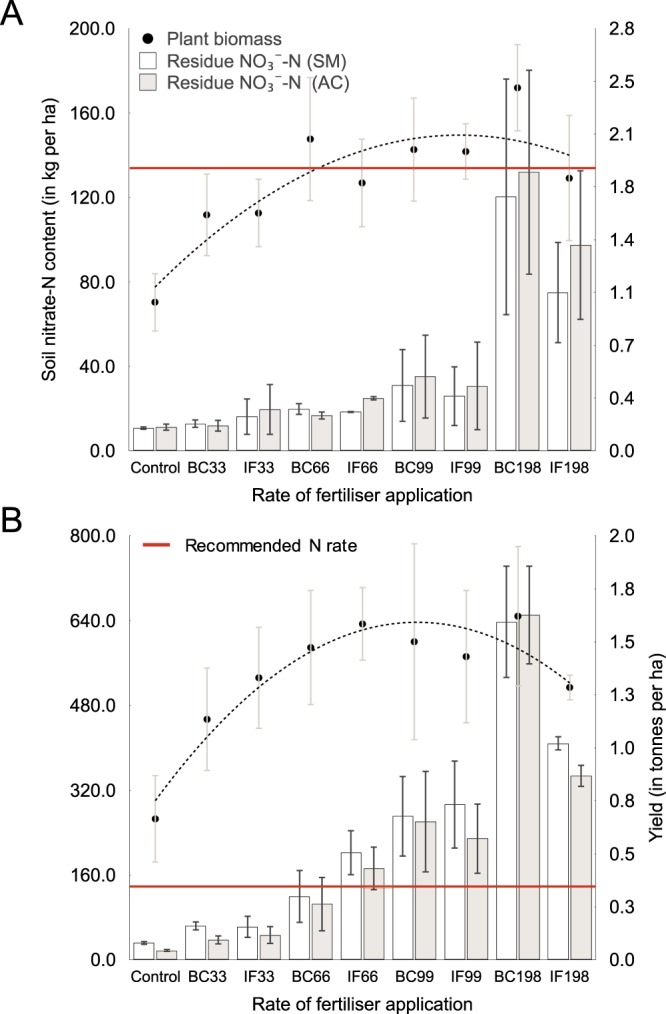
(**A,B**) Water spinach yield was strongly correlated to fertilizer treatment for both biochar (BC) and standard fertilizer (IF) at the rates of 33%, 66%, 99% and 198% of the recommended fertilizer N rate in Trial 1 (**A**) and Trial 2 (**B**). Recommended N rate was obtained from literature. In China, facilitated access to inorganic fertilisers allows farmers to easily reach the recommended quantity of soil N, however, the lack of site-specific soil nutrient status information often leads to overfertilization. The Akvo Caddisfly (AC) app method was applied to assess NO_3_^−^-N level in the soil at harvest (grey bars) alongside the standard method (SM) (white bars). Disparities between the in-field and standard laboratory methods of N assessment were greater at higher NO_3_^−^-N concentrations.

The Akvo Caddisfly method was applied successfully in assessing the level of residue mineral nitrogen (NO_3_^–^N) at harvest (Fig. 3A,B). Measurement of the NO_3_^–^N residues prior to sowing is essential for informing farmers about the potential for nitrate loss due to leaching and denitrification, and the quantity of fertilizer required to be added to subsequent crops, or as a side-dressing, i.e. as intermittent application of fertilizers in a shallow band along the side of a row of crops^3^. Disparities between the in-field and standard laboratory methods of N assessment were found to be greater at higher NO_3_^−^-N concentrations, i.e. for treatments equivalent to two times the recommended fertilizer amount and during the second trial, where the growing conditions were sub-optimal as a result of the less favorable time of the year. In vegetable cultivation, residue nitrogen is likely to be elevated as a result of (1) the crop being harvested prior to achieving maturity, and (2) vegetable residues incorporated into the soil being easily mineralised^5^. In temperate zones, the fall and winter constitute the highest risk period for nitrate leaching from the root zone; in the tropics however, nitrate loss can be independent of the time of the year and has been estimated to be as high as 136 kg ha^−1^ for certain crops in the Chinese greenhouse systems^4,5^. Residual soil nitrate is a good predictor of nitrate leaching loss and as such, the Akvo Caddisfly app provides a valuable support tool for managing this risk.

### Nutrient monitoring across the crop growing season

**Figure 4:**
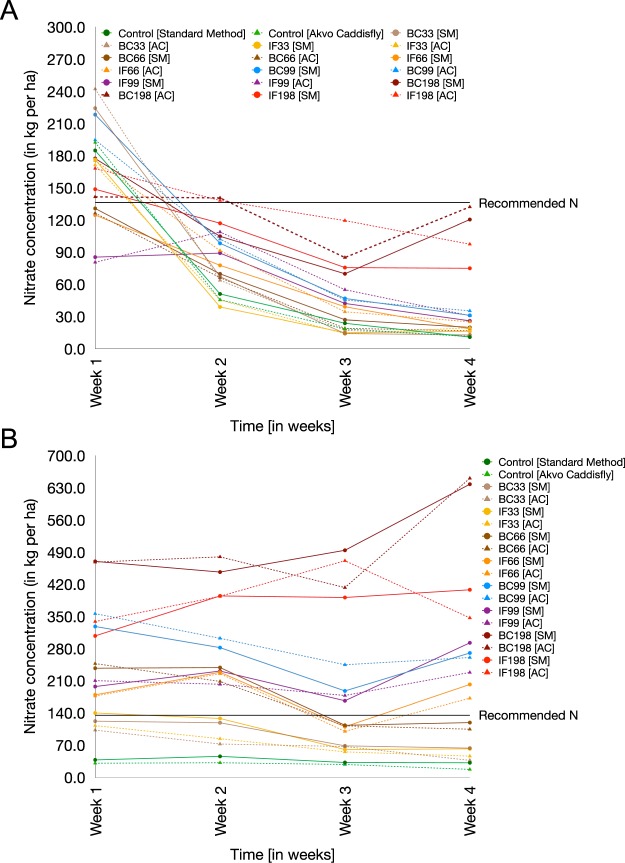
(**A**,**B**) Nitrate-N concentration varied across the crop growing season for Trial 1 (**A**) and Trial 2 (**B**), presented on a weekly basis. The Akvo Caddisfly method was applied to assess NO_3_^−^-N during the plant growth stage. Disparities between the in-field and standard laboratory methods of N assessment were higher during the second trial. Higher quantities of NO_3_^−^-N can be attributed to environmental factors and higher total fertilizer quantity applied.

The Akvo Caddisfly method was found to be capable of determining the quantity of NO_3_^−^-N in the soil throughout the crop growing season (Fig. 4A,B; see Table S1 in Supplementary Material for detailed breakdown of the week-by-week changes in the soil NO_3_^−^-N concentration).

Currently, provision of fertilizers in developing nations can be either subsidized by the respective government, and thus made more affordable, or needs to be purchased with personal resources. Whereas the former situation can lead to overfertilization, the latter calls for optimization of resources to avoid financial losses within vulnerable communities. The ability to monitor changes in nitrate-N concentration across the vegetable growing season allows the farmer to not only fine-tune fertilizer recommendations, but also to improve resource allocation. Chinese farmers have been noted to use as much as 4 670 kg of N ha^−1^ yr^−1 ^^4^, which results in severe soil acidification, nutrient imbalances, heavy metal pollution and abandonment of fields within fifteen years of greenhouse construction^19^. Providing evidence that the soil NO_3_^–^N levels exceed plant requirements might stop the addition of further fertilizer inputs. In contrast, in West Africa, where micro-dosing was proposed as an optimal strategy for increasing crop yield^20^ whilst minimizing expenditure; it would be possible to enhance resource allocation with micro-dosing being implemented on relatively fertile sites within the field, which would then, allow for better management of outfields characterized by lower fertility and erosion risk, e.g. by redirecting manure applications.

The Akvo Caddisfly method was shown to be sensitive enough to track changes in NO_3_^–^N concentrations across the plant growing season. By contrast, soil PO_4_^3−^-P as measured by Akvo Caddisfly and Quantofix test strips revealed limited precision and accuracy. The difference between standard method and Akvo Caddisfly ranged from −62.7 to 57.3 mg kg^−1^ (188.1 to 171.9 kg ha^−1^) for composite samples in Week 1 of the first trial and it was impossible to determine the differences in soil P concentration across the treatments or as the crop season progressed. Phosphate-detecting test strips, as a form of ion chromatography (IC), have been previously shown to be of limited applicability as an agricultural ‘quick test’ in horticultural systems^21^. Similarly, laboratory use of IC has been shown to be a poor measure of extractable P due to multiple interferences and as such is discouraged^22^. However, Akvo Caddisfly showed consistent elevated concentrations of soil P (laboratory range: 231.3 to 341.4 ppm vs. Akvo Caddisfly range: 174.0 to 379.2 ppm), providing useful information regarding overfertilization and over-use of the compound fertilizers that are favored by the local farmers. Other smartphone-mediated soil P tests have been proposed, which do not rely on chromatography^23^ and their continued use should be explored in more detail in similar future studies.

### Uncertainties in soil nutrient estimation with smartphone-mediated soil analysis

**Figure 5:**
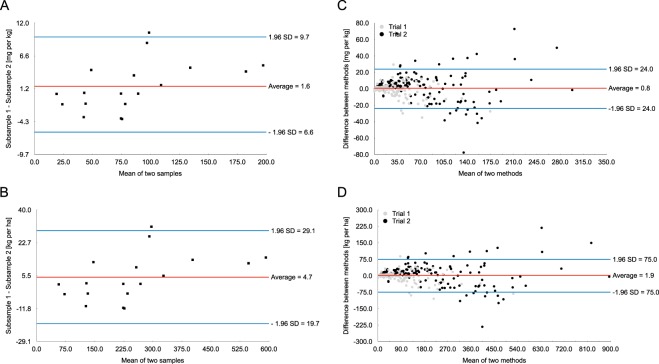
(**A–D**) The subsampling errors measured via the standard laboratory method in mg kg^−1^ (**A**) and kg ha^−1^ (**B**) and errors between smartphone-mediated and standard soil test method for individual measurements expressed in mg kg^−1^ (**C**) and kg ha^−1^ (**D**). The error for soil subsampling i.e. the difference in measurable NO_3_^−^-N resulting from taking only a small portion of the sample for analysis, ranged from −3.8 to 10.4 mg kg^−1^ (19.7 to 29.1 kg ha^−1^). The error range for the difference between smartphone-mediated and standard laboratory NO_3_^−^-N assessment ranged from −24.0 to 24 mg kg^−1^ (−75 to 75 kg ha^−1^). Differences between individual measurements were higher for Trial 2.

The error for soil subsampling defined as the difference in measurable NO_3_^−^-N resulting from taking only a small portion of the sample for analysis, ranged from −3.8 to 10.4 mg kg^−1^ as measured by the autoanalyzer (Fig. 5A). The error range for the difference between the smartphone-mediated and the standard laboratory NO_3_^−^-N assessment was higher than for the soil subsampling error and ranged from −27.1 to 28.4 mg kg^−1^ (Fig. 5B). The difference is likely to be a result of (1) temperature effect on the test strips^24^, (2) chemical interferences^10^, which were more likely to occur at very high fertilizer application rates, and (3) hypothetically, deterioration of the Akvo Caddisfly color correction card. The latter two are likely to be responsible for a greater number of outliers recorded for Trial 2. The deterioration of the color correction card ought to be accounted for if the smartphone-mediated soil test is to be conducted for long periods of time. Similar to the approach proposed by Schmidhalter^25^, it is recommended that a correction factor of 0.2 be used for every 5 °C deviation from the room temperature (approx. 19.5 °C), this having been deemed optimal for the test strip use by the manufacturer [See Figs S1–2 in Supplementary Material for detailed break-down of temperature correction factors]. Addressing the temperature effect is particularly important at higher NO_3_^−^-N concentrations because higher temperature results in large overestimation of readings.

The mean bias (red line) between the subsamples was 1.58 mg kg^−1^, equivalent to 4.70 kg ha^−^, for dry soil samples analyzed with the segmented flow autoanalyzer during a single run of the equipment (Fig. 5A,C). The highest difference recorded for the subsamples ranged from −3.8 to 10.4 mg kg^−1^, equivalent to −11.7 to 31.2 kg ha^−1^ (Fig. 5A,B), with the 95% limits of agreement (expressed as 1.96 × SD) of 9.7 to − 6.6 mg kg^−1^ or 29.1 to −19.7 kg ha^−1^. The mean bias between the standard method and Akvo Caddisfly was 0.80 mg kg^−1^, equivalent to 1.90 kg ha^−1^, for field-moist soil samples (Fig. 5B,D). The highest differences between individual measurements obtained via the standard method and smartphone-mediated soil analysis were −35.0 and 29.5 mg kg^−1^, equivalent to −63.0 to 53.1 kg ha^−1^, for Trial 1 and −77.6 and 72.8 mg kg^−1^, equivalent to −139.7 to 131 kg ha^−1^, for Trial 2 (Fig. 5C,D). The 95% limits of agreement were 24.0 to − 24.0 mg kg^−1^ or 75.0 to −75.0 kg ha^−1^.

Overall, 18%, or 51 out of 284, readings had errors higher or lower than 15 mg kg^−1^ (45 kg ha^−1^), with 43%, or 121 out of 284, readings falling within the error range of −3.6 to 3.8 mg kg^−1^ (11 kg ha^−1^). The highest absolute difference between the methods was recorded for those samples requiring dilution. The same was not found for samples that had to be neutralized with amidosulfuric acid (H_3_NSO_3_) to remove the effect of nitrite inference. Thus, dilution was found to have a disproportionally high impact on the accuracy and precision of readings and thus, as a method should be avoided when possible by e.g. incorporating test strips with a higher range e.g. from 0 to 500 mg kg^−1^ of nitrate as opposed to 0 to 100 mg kg^−1^ as is currently available in the Akvo Caddisfly App. The presence of outliers can be mitigated by taking multiple composite samples across the field. By pooling four measurements across the fields under investigation, the analytical errors were shown to be lower than within-plot differences, thus increasing the quality of the smartphone mediated soil test.

### Field specific soil N level, fertilizer recommendations and cost-savings

Akvo Caddisfly app has been shown to be successful at assessing the requirements for any pre- and in-season N fertilizer applications. Substantial monetary savings can be made by foregoing fertilizer applications in situations where soil N content is already sufficient or exceeds crop needs e.g. where top soil mineral N content is higher than 136 kg of N per ha., as recorded during Trial 2; treatments BC99; BC198; IF99 and IF198 (Table 1). This information could improve nitrogen use efficiency at smallholder farms, reduce associated costs, and lower risks of nitrate leaching to the environment. Quantification of soil N content could form an initial step for introducing a prescriptive-corrective crop nutrient management approach, or for use in discouraging continuous use of compound fertilizers, which has been linked to increased heavy metal concentrations in the soil^19^. Also, whereas soil PO_4_^3−^-P analysis showed limited promise, Kim and Kim^26^ reported successful employment of phosphate test strips to assess total-P level in cucumber (*Cucumis sativus L*.). Akvo Caddisfly offers an opportunity to further examine and expand on those findings by using the App in plant tissue testing study.

**Table 1:** Soil nitrate-N residue calculated based on the standard laboratory analysis (SM) and the smartphone-mediated soil analysis via Akvo Caddisfly (AC) for size of the investigated field (36 m^2^) and 1 ha field together with fertilizer requirements for water spinach and the associated fertilizer costs.

		Soil nitrate-N content^a^						Fertilizer requirement (15:15:15 NPK)^b^				Fertilizer cost (5 CNY per kg)^c^			
		mg per kg		kg per field		kg per ha		kg per field		kg per ha		kg per field		kg per ha	
Treatment		SM	AC	SM	AC	SM	AC	SM	AC	SM	AC	SM	AC	SM	AC
Trial 1	Control	4.3	4.0	0.0	0.0	12.9	12.0	3	3	821	827	15	15	4103	4133
	BC33	6.6	5.6	0.1	0.1	19.8	16.8	3	3	775	795	14	14	3873	3973
	BC66	10.3	11.7	0.1	0.1	30.9	35.1	3	2	701	673	13	12	3503	3363
	BC99	40.1	44.0	0.4	0.5	120.3	132.0	0	0	105	27	2	1	523	133
	BC198	3.6	3.7	0.0	0.0	10.8	11.1	3	3	835	833	15	15	4173	4163
	IF33	5.4	6.5	0.1	0.1	16.2	19.5	3	3	799	777	15	14	3993	3883
	IF66	6.1	8.3	0.1	0.1	18.3	24.9	3	3	785	741	14	13	3923	3703
	IF99	8.6	10.2	0.1	0.1	25.8	30.6	3	3	735	703	13	13	3673	3513
	IF198	25.0	32.5	0.3	0.4	75.0	97.5	1	1	407	257	7	5	2033	1283
		10.8	6.0	0.1	0.1	32.4	18.0	3	3	691	787	13	14	3453	3933
Trial 2	Control	21.4	12.6	0.2	0.1	64.2	37.8	2	2	479	655	9	12	2393	3273
	BC33	40.0	35.2	0.4	0.4	120.0	105.6	0	1	107	203	2	4	533	1013
	BC66	90.4	87.1	1.0	0.9	271.2	261.3	Overfertilization & Overspending							
	BC99	212.6	216.9	2.3	2.3	637.8	650.7								
	BC198	20.9	15.6	0.2	0.2	62.7	46.8	2	2	489	595	9	11	2443	2973
	IF33	67.5	57.6	0.7	0.6	202.5	172.8	Overfertilization & Overspending							
	IF66	97.7	76.3	1.1	0.8	293.1	228.9								
	IF99	136.1	115.8	1.5	1.3	408.3	347.4								
	IF198	4.3	4.0	0.0	0.0	12.9	12.0	3	3	821	827	15	15	4103	4133

^a^The average concentration of nitrate-nitrogen measured across four plots; ^b^Recommended rate of nitrogen application for I. aquatica is equal to 136 kg of N per ha; ^c^The price of 1 kg of 15:15:15 NPK compound inorganic fertilizer (136 kg of N = 906 kg of 15% N inorganic fertilizer) in rural Jiangsu Province, China.

It is important to note that the soil organic matter and the soil’s capacity for N mineralization is not accounted for by Akvo Caddisfly currently. However, a smartphone application for assessment of soil organic matter content has already been developed^23^, but is to date restricted to those countries with well-developed national soil databases. In the future, the lab-on-a-chip approach might integrate multiple smartphone apps, which can act as decision support tools, to address shortcomings of and further improve available technological solutions.

Overall, optimizing fertilizer utilization rates without *a priori* knowledge of soil conditions constitutes a two-pronged challenge. Firstly, application of insufficient quantities of fertilizer results in diminished returns on investment, especially in places where fertilizers are expensive and non-subsidized^7^. Secondly, and conversely, applying it in excessive amounts leads to environmental pollution and mineral nutrient imbalances that negatively affect crop yields^27^ and ultimately the sustainable productivity of the land, as well as unnecessary costs being borne. Considering the rate of environmental degradation and growing human population, it is crucial to commence the shift of the farming systems towards agriculture that is both efficient, smart and sustainable^28^. As the use of Big Data^29^ and technologies such as remote sensing^30^, robotics^31^, and non-destructive soil and plant tissue testing^8^ are being increasingly embraced; it is essential to ensure that access to agricultural decision support tools is made affordable to all interested parties. Smartphones offer a promising future for development of relatively inexpensive and user-friendly support tools for agricultural systems^32^, which might prove to be easily accessible to agricultural workers across the world.

## Conclusions

This paper has investigated the potential for employing a smartphone app, Akvo Caddisfly, together with nitrate- and phosphate-sensitive test strips used to assess the content of plant available nutrients in the soil. The results have indicated that smartphone-mediated soil analysis can be successfully conducted for NO_3_^−^-N, but that there is currently only limited success with accurate assessment of soil PO_4_^3−^-P content. Analytical errors associated with the in-field nutrient analyzer can be minimized by taking multiple composite samples across the field, ensuring optimal light conditions, accounting for temperature effects, and increasing the number of test strips used per sample. Regardless of shortcomings, such as temperature dependency, chemical interferences and decreased accuracy at high nutrient concentration, this approach has the potential to provide a useful fertilizer recommendation tool in circumstances where access to conventional soil testing methods is limited. Further studies should involve investigation of ammonia test strips (currently incorporated into the Akvo Caddisfly) that showed promise during initial trials, and application of smartphones and test strips in plant sap measurement, to better inform agricultural management decisions at local level. Overall, employing smartphone technology, alongside local agronomic knowledge, has great potential for democratizing access to field-scale soil fertility data and improving sustainable fertilizer management throughout the world.

## Supplementary information

Additional information to support main dataset
