# Supplementary material for: The potential for using smartphones as portable soil nutrient analyzers on suburban farms in central East China
Source: Sci Rep. 2019 Nov 11;9:16424. doi: 10.1038/s41598-019-52702-8 (PMC6848085; doi:10.1038/s41598-019-52702-8)
Supplement: Supplementary file 1 — Additional information to support main dataset [file 41598_2019_52702_MOESM1_ESM.doc]

**The potential for using smartphones as portable soil nutrient analyzers on suburban farms in central East China**

Karolina Golicz^1^, Stephen H. Hallett^1^, Ruben Sakrabani^1*^, Genxing Pan^2^

^1^School of Water, Energy and Environment, Cranfield University, Cranfield, Bedfordshire MK43 0AL, UK

^2^ Department of Soil Science and Institute of Resource, Ecosystem and Environment of Agriculture, Nanjing Agricultural University, 1 Weigang, Nanjing 210095-China

*Corresponding author : [r.sakrabani@cranfield.ac.uk](mailto:r.sakrabani@cranfield.ac.uk)

**SUPPLENTARY MATERIAL**

1. **Correction equation to calibrate Akvo Caddisfly results to standard methods of Nitrate-N and P analyses used in the UK.**

1. **Temperature correction factors for NO_3_¯ (SFig 1.) and PO_4_^3^¯ (SFig. 2) Quantofix test strips.**

| 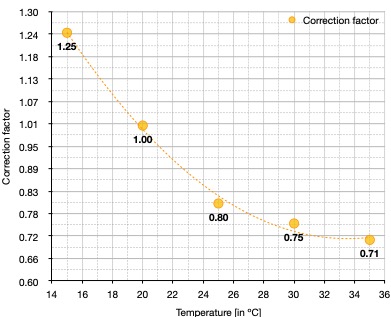 |
| --- |
| **Fig S1.** Correction factors developed for nitrate test strips through Quantofix Relax to account for temperature dependency. Temperatures investigated comprised: 15, 20, 25, 30, 35 °C, at a humidity of 70%. The study was conducted in a temperature-controlled plant growth chamber at Cranfield University. |
|  |
| **Fig S2.** Correction factors developed for phosphate test strips through Quantofix Relax to account for temperature dependency. Investigated temperatures constituted: 15, 20, 25, 30, 35 °C, at humidity of 70%. The study was conducted in a temperature-controlled plant growth chamber at Cranfield University. |

3. Detailed account of soil nitrate-N concentration (Table S1).

| Table S1. Comparison of soil nitrate-N level across the crop growing trials (N=4). | | | | | | | | | | | |
| --- | --- | --- | --- | --- | --- | --- | --- | --- | --- | --- | --- |
|  |  | |  | **TRIAL 1** | | | | **TRIAL 2** | | | |
|  |  |  |  | **Autoanalyzer** | | **Akvo Caddisfly** | | **Autoanalyzer** | | **Akvo Caddisfly** | |
| **Week** | **Treatment** | **Fertilizer type** | **N** | **Mean** | **SD** | **Mean** | **SD** | **Mean** | **SD** | **Mean** | **SD** |
| 1 | 0 | Control | 4 | 184.7 | 192.2 | 148.1 | 179.2 | 38.6 | 7.5 | 31.4 | 13.2 |
| 1 | 0.33 | Biochar | 4 | 224.0 | 241.9 | 177.1 | 221.2 | 122.9 | 24.8 | 103.4 | 21.6 |
| 1 | 0.66 | Biochar | 4 | 130.6 | 126.2 | 50.9 | 47.1 | 237.9 | 40.0 | 248.0 | 101.2 |
| 1 | 0.99 | Biochar | 4 | 218.0 | 194.5 | 70.3 | 81.3 | 328.7 | 77.7 | 356.7 | 125.6 |
| 1 | 1.98 | Biochar | 4 | 177.2 | 141.4 | 55.0 | 59.1 | 470.2 | 189.7 | 468.5 | 125.2 |
| 1 | 0.33 | Inorganic | 4 | 176.2 | 171.2 | 89.6 | 119.3 | 140.8 | 29.5 | 113.3 | 34.4 |
| 1 | 0.66 | Inorganic | 4 | 124.4 | 175.2 | 24.2 | 111.3 | 180.0 | 29.9 | 177.5 | 66.7 |
| 1 | 0.99 | Inorganic | 4 | 85.3 | 80.5 | 25.4 | 38.6 | 198.0 | 53.5 | 210.9 | 60.6 |
| 1 | 1.98 | Inorganic | 4 | 148.5 | 167.9 | 91.4 | 106.6 | 308.3 | 42.9 | 339.5 | 68.1 |
|  |  |  |  |  |  |  |  |  |  |  |  |
| 2 | 0 | Control | 4 | 50.9 | 45.5 | 28.9 | 29.2 | 46.4 | 17.9 | 32.5 | 20.3 |
| 2 | 0.33 | Biochar | 4 | 67.1 | 63.7 | 25.5 | 28.3 | 119.5 | 33.6 | 73.2 | 18.2 |
| 2 | 0.66 | Biochar | 4 | 69.7 | 66.1 | 28.7 | 30.5 | 239.4 | 128.2 | 209.2 | 130.4 |
| 2 | 0.99 | Biochar | 4 | 98.2 | 102.1 | 44.4 | 48.0 | 282.8 | 67.3 | 303.1 | 98.8 |
| 2 | 1.98 | Biochar | 4 | 104.7 | 140.4 | 26.9 | 41.3 | 446.9 | 71.0 | 480.0 | 98.8 |
| 2 | 0.33 | Inorganic | 4 | 38.8 | 45.2 | 35.2 | 45.1 | 128.9 | 25.5 | 84.8 | 20.1 |
| 2 | 0.66 | Inorganic | 4 | 77.6 | 91.3 | 19.7 | 28.2 | 229.1 | 76.0 | 226.1 | 93.1 |
| 2 | 0.99 | Inorganic | 4 | 89.0 | 108.8 | 55.4 | 81.7 | 231.8 | 11.2 | 203.0 | 54.6 |
| 2 | 1.98 | Inorganic | 4 | 116.8 | 138.0 | 70.3 | 97.5 | 395.0 | 56.2 | 394.2 | 48.8 |
|  |  |  |  |  |  |  |  |  |  |  |  |
| 3 | 0 | Control | 4 | 23.7 | 18.5 | 21.5 | 17.2 | 33.0 | 8.7 | 28.7 | 12.8 |
| 3 | 0.33 | Biochar | 4 | 14.1 | 17.2 | 13.1 | 11.0 | 69.2 | 33.0 | 67.8 | 20.4 |
| 3 | 0.66 | Biochar | 4 | 26.9 | 19.0 | 10.2 | 12.4 | 114.0 | 28.7 | 112.8 | 45.7 |
| 3 | 0.99 | Biochar | 4 | 46.7 | 45.2 | 26.3 | 29.2 | 188.5 | 69.4 | 245.5 | 186.0 |
| 3 | 1.98 | Biochar | 4 | 69.8 | 84.9 | 47.9 | 66.5 | 494.3 | 385.4 | 413.2 | 283.7 |
| 3 | 0.33 | Inorganic | 4 | 14.9 | 14.0 | 12.2 | 12.7 | 61.2 | 34.1 | 56.1 | 19.8 |
| 3 | 0.66 | Inorganic | 4 | 39.0 | 34.3 | 22.7 | 18.1 | 110.8 | 27.2 | 100.7 | 27.9 |
| 3 | 0.99 | Inorganic | 4 | 42.1 | 54.9 | 37.1 | 59.4 | 167.1 | 104.7 | 178.6 | 131.6 |
| 3 | 1.98 | Inorganic | 4 | 75.5 | 119.3 | 65.2 | 119.5 | 391.6 | 43.9 | 471.6 | 52.1 |
|  |  |  |  |  |  |  |  |  |  |  |  |
| 4 | 0 | Control | 4 | 10.8 | 11.2 | 1.6 | 3.3 | 32.4 | 8.0 | 18.0 | 6.7 |
| 4 | 0.33 | Biochar | 4 | 12.8 | 11.9 | 4.1 | 5.8 | 64.3 | 16.9 | 37.7 | 17.7 |
| 4 | 0.66 | Biochar | 4 | 19.9 | 16.7 | 5.8 | 3.9 | 119.9 | 100.7 | 105.5 | 102.7 |
| 4 | 0.99 | Biochar | 4 | 31.0 | 35.2 | 34.6 | 39.7 | 271.1 | 152.3 | 261.2 | 192.3 |
| 4 | 1.98 | Biochar | 4 | 85.1 | 132.0 | 49.7 | 97.3 | 637.9 | 212.7 | 650.8 | 186.9 |
| 4 | 0.33 | Inorganic | 4 | 16.2 | 19.6 | 17.4 | 24.2 | 62.7 | 42.4 | 46.9 | 34.1 |
| 4 | 0.66 | Inorganic | 4 | 18.4 | 25.0 | 0.9 | 2.1 | 202.6 | 85.7 | 172.7 | 82.7 |
| 4 | 0.99 | Inorganic | 4 | 25.9 | 30.7 | 28.6 | 42.2 | 293.2 | 166.3 | 228.8 | 132.4 |
| 4 | 1.98 | Inorganic | 4 | 74.9 | 97.4 | 48.1 | 71.0 | 408.4 | 27.7 | 347.5 | 42.4 |
